# Redescription of *Platygyndes* Roewer 1943, a false Gonyleptidae, (Arachnida, Opiliones, Cosmetidae)

**DOI:** 10.3897/zookeys.143.1916

**Published:** 2011-11-01

**Authors:** Ricardo Pinto-Da-Rocha, Marcos Ryotaro Hara

**Affiliations:** 1 Departamento de Zoologia, Instituto de Biociências, Universidade de São Paulo, Caixa Postal 11461, São Paulo, SP, Brazil, 05422-970; 2 Escola de Artes, Ciências e Humanidades, Universidade de São Paulo, Av. Arlindo Bettio no 1000, Ermelino Matarazzo, São Paulo, SP, Brazil, 03828-000

**Keywords:** Andes, Neotropical fauna, systematics, taxonomy, harvestmen

## Abstract

*Praelibitia* Roewer, 1956 and its type species, *Praelibitia titicaca* Roewer, 1956, are respectively synonymized with *Platygyndes* Roewer, 1943 and its type species *Platygyndes titicaca* Roewer, 1943, and furthermore the genus is transferred from the Gonyleptidae to the Cosmetidae. On the basis of domed and unarmed ocularium, increased number of granules on scutal areas, unarmed dorsal scutum and general body shape, *Platygyndes* seems to be closely related to *Moselabius* Roewer, 1956 and *Caracarana* Roewer, 1956. External morphological characters that are useful to revealing relationships among cosmetid genera are discussed.

## Introduction

Cosmetidae is a highly diverse Laniatores family with over 700 described species distributed from southern USA to southern Patagonia, including the Greater and Lesser Antilles (Kury 2003; Pinto-da-Rocha and Kury 2007; Hallan 2011). The family is easily diagnosed by the pedipalps, which cover the frontal part of the chelicerae: pedipalpal femur is strongly compressed laterally, pedipalpal tibia is spoon-shaped and unarmed or weakly armed (Kury and Pinto-da-Rocha 2007). Despite the impressive richness (the third most speciose family of Opiliones), only a few attempts, such as Ferreira and Kury (2010), have been made to review its classification using alternative characters compared to those used by Rower. Some recent works, e.g. Townsend et al. (2010), are only an extension of Roewer’s system, enriched by images of male genitalia, of which characters were not used in the generic classification. Other rich Neotropic families, such as the Sclerosomatidae and the Gonyleptidae, have received much more attention in the last decade. Several subfamilies of the Gonyleptidae have been revised (Pinto-da-Rocha 2002; Yamaguti and Pinto-da-Rocha 2009; DaSilva and Gnaspini 2009; DaSilva and Pinto-da-Rocha 2010; Pinto-da-Rocha and Bragagnolo 2010), as well as the South American Sclerosomatidae (Tourinho and Kury 2001, 2003; Tourinho 2003, 2004a, 2004b). Yet, the Cosmetidae remains the biggest challenge for the laniatorid systematics of the 21^st^ century.

This paper is a first step in tackling this challenge, and it is based on our current investigations relevant to a revision of the Gonyleptidae. Here we redescribe *Platygyndes titicaca* Roewer, 1943, so far considered in the Gonyleptidae (Pachylinae), and propose its transfer to the Cosmetidae. Indeed, this species possesses some features unusual for the cosmetids: viz., (i) pedipalpal femur is moderately flattened and not projected dorsally; (ii) pedipalpal tibia is moderately flattened laterally; and (iii) evident scutal grooves I–V present. It is possible that these features would have misled Roewer who placed *Platygyndes* in the Gonyleptidae. Finally, based on its external morphology, *Platygyndes titicaca* Roewer, 1943 is found to be a senior synonym of *Praelibitia titicaca* Roewer, 1956; it is another example of the same species described by Roewer twice, with its male and female placed in different families.

## Methods

The nomenclature follows Acosta et al. (2007), with some modifications adapted specifically to the studied group. Carapace refers to the part of dorsal scutum that covers the prosoma. The scutal area V is referred to as posterior margin of dorsal scutum. SMF stands for the depository of Naturmuseum Senckenberg, Frankfurt am Main, Germany (curator: P. Jäger). In synonymic lists, we adopted the following abbreviations: cat=catalogue; juv=juvenile(s); rdes=redescription. Only the different characteristics regarding the male were mentioned in the female redescription. The illustrations of the external morphology were made under a stereomicroscope using camera lucida with the material immersed in 70% ethanol. The genitalia were prepared according to Pinto-da-Rocha (1997) and illustrated using a compound microscope with camera lucida. Measurements are in millimeters.

We have also examined the type materials of ten laniatorid species from the genera which may be phylogenetically close to *Platygyndes*: *Eulibitia annulipes* Roewer, 1912 (male holotype; SMF 447); *Eulibitia maculata* Roewer, 1912 (male holotype; SMF 471); *Eulibitia sexpunctata* Roewer, 1919 (male holotype; SMF 473); *Caracarana inermis* Roewer, 1956 (male holotype; SMF 9730); *Metalibitia adunca* (Roewer, 1927) (male holotype; SMF 143/9); *Metalibitia borelli* (Roewer, 1925) (male paratype; SMF 121/3); *Metalibitia maculata* (Roewer, 1914) (1 male and 1 female paratypes; SMF 1060); *Metalibitia tibialis* (Roewer, 1925) (2 males; SMF 122/4); *Moselabius albipunctatus* Roewer, 1956 (6 males and 14 females; SMF 1394/297); *Syncynorta longipes* Roewer, 1947 (female holotype; SMF 5865/207).

## Systematics

### Cosmetidae, Cosmetinae

#### Platygyndes

Roewer, 1943 new familial and subfamilial assignment

http://species-id.net/wiki/Platygyndes

Platygyndes Roewer, 1943: 16; Soares and Soares 1954: 291 (rdes, cat). Type species *Platygyndes titicaca* Roewer, 1943, by monotypy.
Praelibitia Roewer, 1956: 442 (type species *Praelibitia titicaca* Roewer, 1956, by original designation). new synonymy.

##### Diagnosis.

*Platygyndes* is a Cosmetidae having the domed and narrow ocularium, instead of the depressed medially and widened one, which is common in eastern and several Andean species. Moreover, this genus possesses the well-marked scutal grooves I–V; the moderately flattened, not dorsally projected pedipalpal femur; and the moderated, laterally flattened pedipalpal tibia which strongly contrast with the typical type observed in the family (strongly flattened and spoon-shaped). On the basis of unarmed domed ocularium and dorsal scutum, the genus seems to be more closely related with *Moselabius* Roewer, 1956 and *Caracarana* Roewer, 1956. *Moselabius* known only after a female can be distinguished from *Platygyndes* by larger and sparser tubercles on the dorsal scutum and a paramedian pair of the enlarged tubercles on free tergites I–III and thickened tibiae IV. *Caracarana* differs from *Platygyndes* by the incrassate femur IV, the pedipalpal tibia with a ventral projection, the thickened and basally constricted tibia IV, the thickened and curved metatarsus IV and the long tarsal process.

#### Platygyndes titicaca

Roewer, 1943

http://species-id.net/wiki/Platygyndes_titicaca

Figs 1 [Fig F2] 3

Platygyndes titicaca Roewer, 1943: 16, pl. 1, fig 1; Soares and Soares 1954: 291 (cat); Acosta 1996: 222 (cat); Kury 2003: 187 (cat) (Peru [“Titicaca Seeufer”], male holotype, SMF RII 7736/112, examined).
Praelibitia titicaca Roewer, 1956: 442; Kury 2003: 82 (cat) (Peru [“bei Chucuito am Titicaca See, 3900 m”], female holotype, 11.III.53, H.W. Koepcke leg., SMF RII 9726; idem, 1 female & 4 juv. paratypes [however only 1 female & 2 juv. in vial], SMF 9727, examined). NEW SYNONYMY

##### Type locality.

Peru: Puno (shores of Titicaca Lake).

##### Note.

The label of type material of *Platygyndes titicaca* has no data beyond “Titicaca-Seeufer” (shores of Titicaca Lake), although Roewer (1943) clearly states Peru as the type locality of this species. Kury (2003) argued that the department of Puno (the sole department close to Titicaca Lake in the Peruvian side) seems to be a more precise type locality of the species; he also indicated that the correct country could be Bolivia. We agree with the latter suggestion of Kury (2003).

##### Material examined.

PERU. Puno: without further data on locality (“Titicaca Seeufer” [shores of Titicaca Lake]), male holotype of *Platygyndes titicaca*, without more precise locality, name of collectors or date, SMF RII 7736/112; Chucuito (“bei Chucuito am Titicaca See” [near Chucuito at Titicaca Lake], 3900 m), female holotype of *Praelibitia titicaca*, 11.III.53, H.W. Koepcke leg., SMF RII 9726; idem, 1 female & 2 juv. paratypes of *Praelibitia titicaca*, SMF RII 9727.

##### Description.

**Figure 1.**
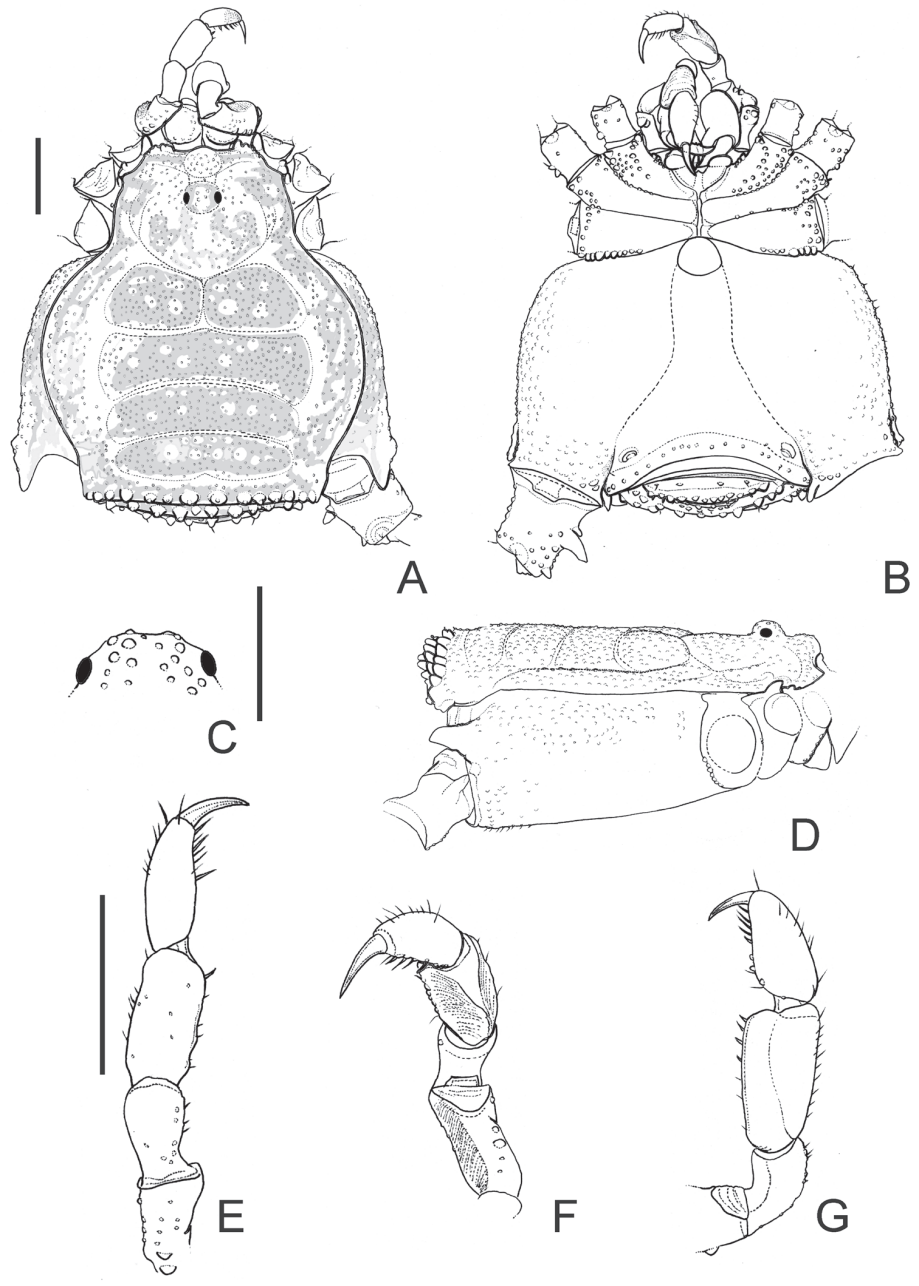
*Platygyndes titicaca* Roewer. Male (holotype): **A** habitus, dorsal view **B** ditto, ventral view **D** ditto, right lateral view **C** ocularium, anterior view **E** left pedipalp, dorsal view **F** ditto, ventral view **G** retrolateral view **A, B, D** at the same scale **E–G** at the same scale. Scale bar 1 mm except for **C** which is 0.5 mm.

**Figure 2. F2:**
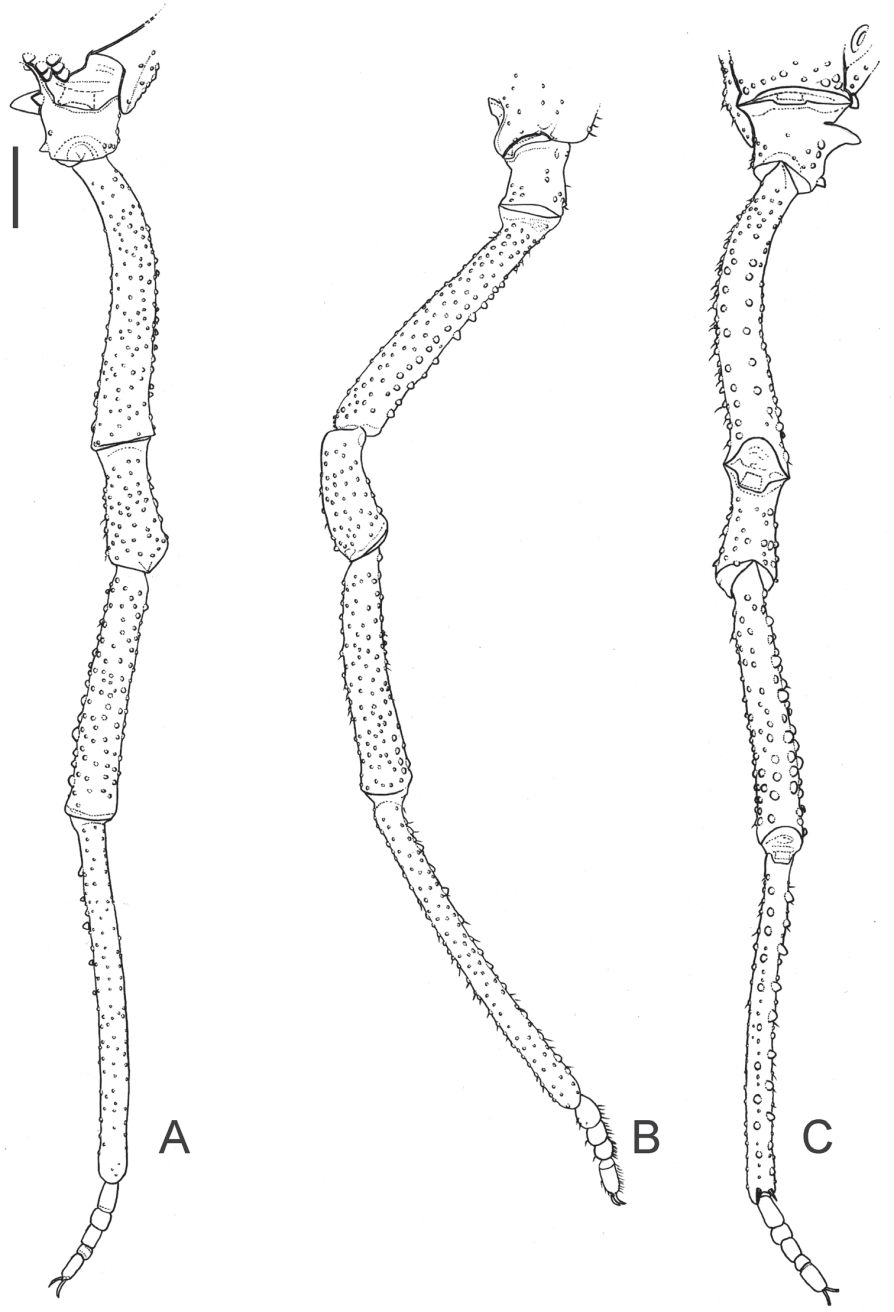
*Platygyndes titicaca* Roewer. Male (holotype): Right leg IV **A** dorsal view **B**, prolateral view **C** ventral view. Scale bar: 1 mm.

**Figure 3.**
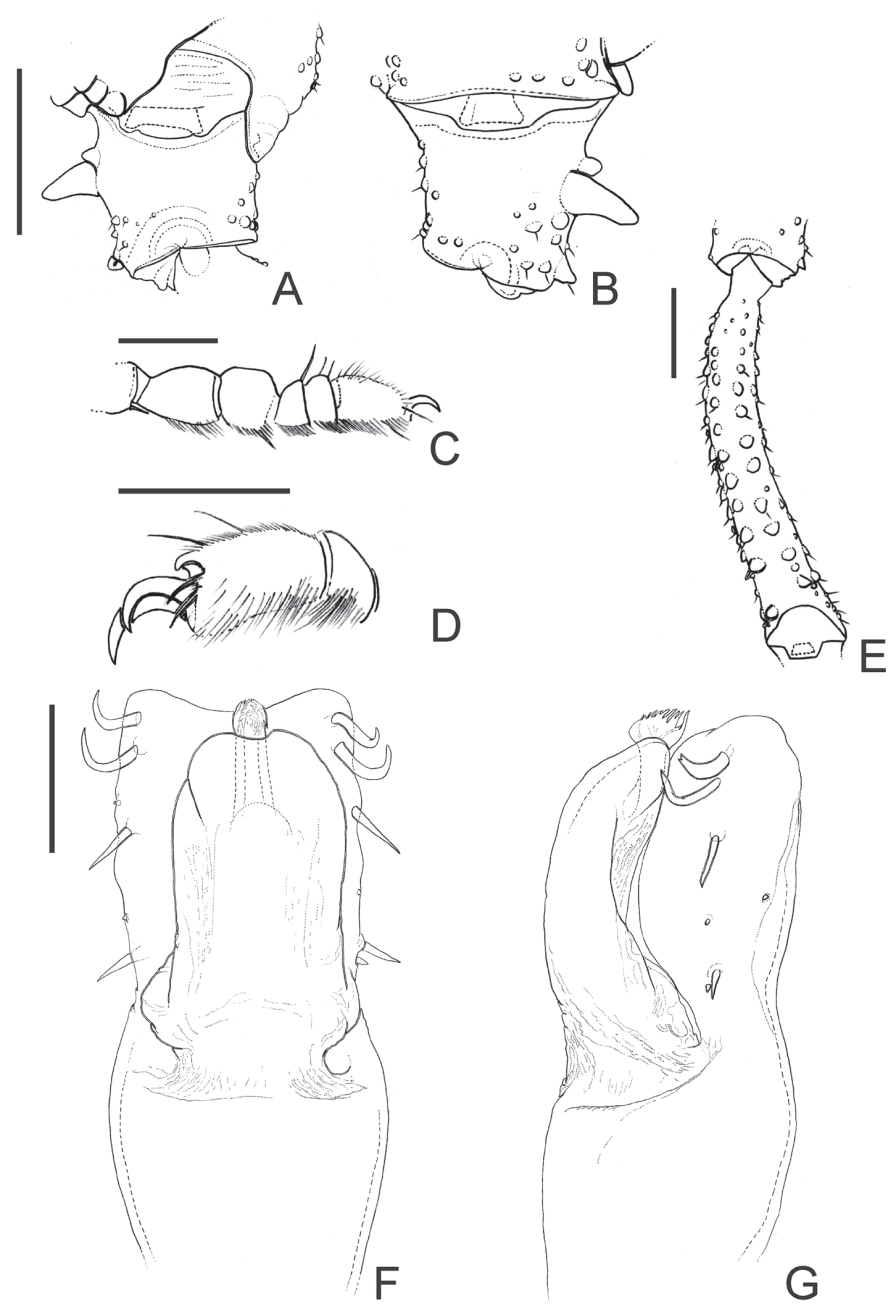
*Platygyndes titicaca* Roewer. Male (holotype): **A** right trochanter IV, dorsal view **B** ditto, ventral view **C** right tarsomeres I, retrolateral view **D** distalmost right tarsomere IV, prolateral view **E** right femur III, ventral view **F** penis, dorsal view **G** ditto, right lateral view **A, B** at the same scale **F–G** at the same scale. Scale bars **A–E** 1 mm **F, G** 0.1 mm.

Male (holotype; SMF RII 7736/112). Measurements: carapace maximum length 1.8; carapace maximum width 2.1; dorsal scutum maximum length 4.6; dorsal scutum maximum width 4.1; femur IV length 3.1; legs I–IV length 6.8; 10.9; 9.9; 13.9. Dorsum (Fig. 1A, C, D): dorsal scutum shape type gamma (Kury et al. 2007), flattened, granulated, widest at scutal area II. Paracheliceral projections not conspicuous, rounded. Anterior margin of dorsal scutum with three enlarged and fused together tubercles on each corner. Ocularium domed (without median depression), narrow (around a fifth of carapace width), densely minute-tuberculate. Lateral margin of dorsal scutum with less granules than scutal areas. Scutal grooves I–V clearly visible, delimiting four scutal areas. Scutal areas I–IV unarmed, I divided by a longitudinal groove. Posterior margin of dorsal scutum with a row of 14 conical, enlarged tubercles. Free tergites I–III granulated, each with a row of 11, 9 and 10 conical, enlarged minute tubercles, respectively. Anal opercle with anterior row of 6 and a group of 16 tubercles. Venter (Fig. 1B): coxae I–IV granulated, distal half of coxae I, distal posterior of coxae II–III with enlarged tubercles. Posterior margin fused to the stigmatic area slightly concave. Mesotergal sternites each with a row of minute tubercles. Anal opercle with one anterior and one posterior row of tubercles. Chelicera (Fig. 1A): not swollen. Bulla dorsally covered by tubercles. Movable and fixed fingers each with 4 tooth. Pedipalps (Fig. 1E–G): trochanter with three ventral tubercles. Femur moderately flattened, not projected dorsally, with five dorsal wide tubercles, four ventral tubercles (subdistal one largest). Tibia spatulate, moderately projected ventrally, tibia–tarsus with lateral setae. Legs (Figs 1A, 2, 3A–E): coxa I with one prolateral apophysis, this blunt, large and one retrolateral bifid apophysis; II with one prolateral apophysis, this large, obliterating ozopore and curved frontwards and one retrolateral apophysis, this fused with prolateral apophysis of coxa III; III with one prolateral, one retrolateral apophyses; IV anteriorly with a shoulder-like shape in dorsal view, reaching scutal groove IV, densely granulated, one prolateral apical apophysis with capitate apex directed backwards, one retrolateral apical large tubercle. Trochanters I–IV granulate; I–II with two retrolateral enlarged tubercles; III with one retrolateral enlarged tubercle; IV retrolaterally with a median apophysis, this conical, its length half of the podomere width, one submedian and one apical enlarged tubercles. Femora and tibiae I–IV tuberculate and roughly arranged in longitudinal rows. Femora III–IV slightly curved, with two ventral rows of tubercles slightly increasing in size apicad, more conspicuous in femur III. Tibia–metatarsus IV ventrally with enlarged tubercles. Tarsi I with globose and short tarsomeres; III–IV with smooth claws, short tarsal process (around a fifth of tarsal claw length). Tarsal formula: 5(3), 5–6(3), 5, 5. Penis (Fig. 3F, G): glans elongated, covering most of stylus dorsally. Stylus with inflated apex and thin projections in distal margin dorsoventrally. Ventral plate rectangular, thick, with two pairs of curved distal setae, one pair of straight submedian setae, two pairs of basal setae (the basalmost one shortest), two pairs of very small setae (placed between the main groups of setae on the left or between submedian and basal group of setae on the right).

Female (holotype of *Praelibitia titicaca*; SMF RII 9726). Measurements: carapace maximum length 1.8.; carapace maximum width 2.0; dorsal scutum maximum length 5.2; dorsal scutum maximum width 4.3; femur IV length 3.1; leg I–IV length 7.2; 10.9; 9.4; 12.9. Dorsum: dorsal scutum shape type alpha, wider at scutal groove II, narrowed at scutal area III. Posterior margin of dorsal scutum and free tergites I–III each with a row of 13, 9, 11 and 10 conical, enlarged tubercles, respectively. Legs: coxa IV only visible apically (in dorsal view), reaching groove III, with prolateral apical apophysis shorter than male. Trochanters I–IV without enlarged tubercles or apophyses. Femur and tibia–metatarsus IV ventrally with tubercles of similar size. Tarsal formula: 5(3), 5(3), 5, 5.

##### Remarks.

*Platygyndes titicaca* possesses the moderately flattened pedipalpal femora which are not projected dorsally and the moderated, laterally flattened pedipalpal tibia compared to the spoon-shaped (flattened and concave) in the majority of cosmetids; besides, it has the unusually well-marked scutal grooves I–V. These unusual features might have led Roewer to assign it to the Gonyleptidae, Pachylinae. It is worth mentioning that the male genitalia of *Platygyndes titicaca* are undoubtedly of the cosmetid groundplan, not of that of the Gonyleptidae. We have examined the Andean material deposited in the SMF and realized that *Praelibitia titicaca* described from a female was also collected close to the type locality of the monotypic *Platygyndes*. Considering the sexual dimorphism in cosmetids, the general body shape, ocularium and pedipalpal shape, dorsal scutum ornamentation and the remaining colour pattern, we have conclude that both names are synonyms. Thus the same species was classified by Roewer in different families, indicating once more that the Roewerian system of Opiliones is hardly reliable.

## Discussion

Due to poor taxonomic characterization of the genera included in Cosmetidae, this family is among the least understood Neotropical Laniatores. Most genera are still diagnosed by the Roewerian combination of armature on dorsal scutum and number of tarsal segments (e.g., Townsend et al. 2010). The Roewerian system relied on a limited set of characters and therefore highlighted predominantly the differences among species/specimens and resulted in many artificial groupings and monotypic genera. In the 1950s, certain attempts to resolve this situation were undertaken by the authors (e.g., Mello-Leitão 1945, Soares 1943, Soares and Soares 1954) who began to take into account an intraspecific variation. In the Cosmetidae, for instance, Goodnight and Goodnight (1953) proposed synonymies for many genera, arguing that the observed differences were due to an intraspecific variation. Such tendency to synonymize the opilionid taxa described by Roewer had lasted until the end of the XX century, although in a more argumentative way.

Recently, Kury et al. (2007) advocated in using characters of the dorsal scutum. In *Platygyndes*, the shape of dorsal scutum is similar to the gamma-type: viz., the scutum convexity is much wider and displaced posteriorly, and there is a well-marked anterior constriction (as in *Metalibitia*) (see Table 1). However, the posterior constriction is well-marked as well, differing from the original definition and being more similar to the alpha-type (see Kury et al. 2007). Townsend et al. (2010) also reported on difficulties in classifying the alpha or gamma types. Having examined the female of *Platygyndes titicaca*, we can confirm that its dorsal scutum shape is clearly of the alpha-type. These data suggest that the shape of dorsal scutum may vary due to sexual dimorphism and such intraspecific variation should be considered while classifying its shape. Thus, we are of the opinion that the alpha-type should also include those dorsal scuta which present a well-marked posterior constriction and the strikingly widened part at rear. A practical option would be just merging the alpha- and gamma-types in a single category. Another structure that seems to be useful in delimitating genera is the male coxae IV: viz., its length (reaching the grooves III, IV, or the posterior margin of dorsal scutum), its visible extension in dorsal view (hidden or not under the dorsal scutum), its shape (parallel or apically divergent) and its apical armature. Additionally, the dorsal and ventral armature of tubercles on pedipalpal femora and the shape of pedipalpal tibia are also useful for a generic delimitation.

We have also found the ocularium to be very informative, although overlooked by many authors. Having examined the cosmetids from Andes and the eastern part of South America, we have confirmed the existence of at least two very distinct types of the ocularium: (i) the widened and medially depressed one, as in *Cynorta* (Kury et al. 2007, fig. 1), a condition which is indeed typical for the majority of cosmetids (Kury and Pinto-da-Rocha 2007; Ferreira and Kury 2010); and (ii) the narrow and domed one, as in *Platygyndes* (see Fig. 1A) and *Caracarana*. On the basis of the similarity both of the body shape and of the narrow ocularium, we consider *Metalibitia*, *Eulibitia*, *Moselabius* and *Syncynorta* as likely to be closely related to *Platygyndes* (Table 1); yet those genera possess the depressed ocularium. Furthermore, the pedipalp considered to date as a very conservative character of the family seems to be also useful for phylogenetic assessments. *Platygyndes* stands out as a cosmetid genus with moderate modifications of its pedipalps: viz., of femora and tibia, which are moderately flattened instead of strongly flattened laterally, and the typical spoon-shaped tibia are poorly-marked (this is why Roewer should have assigned this genus in the Gonyleptidae). Reasoning from the fact that cosmetid juveniles possess cylindrical pedipalps before gaining it of the typical shape in adults, it is safe to conclude that *Platygyndes* may belong to a basal cosmetid lineage in which the spoon-shaped tibia are fully developed.

The aforementioned characters are likely to be useful in revealing phylogenetic relationships within the speciose Cosmetidae. A high number of the described species, as well as their poor descriptions and illustrations, and past failures in resolving the taxonomic status of confusing species and genera definitely present a serious challenge (even more serious than that posed by the Gonyleptidae 20 years ago). Although being time and resource consuming, the most reliable option to tackle this challenger seems to be a re-examination of available type material and a further search for reliable characters in order to better resolve phylogenetic relationships within the family.

**Table 1.** Comparison of the genera of Cosmetidae with narrow ocularium. Body shape according to Kury et al. (2007). ? = refers to unknown male.

Genera	Body shape	Male coxa IV	Ocularium shape	Body dorsal armature	Dorsal pedipalpal tibia shape	Dimorphic chelicera
*Platygyndes*	γ (male), α (female)	Entirely visible in dorsal view, reaching groove IV	Domed	Scutal areas unarmed; posterior margin of dorsal scutum and free tergites with a row of conical, enlarged tubercles	Almost rectangular	Absent
*Caracarana*	α	Entirely visible in dorsal view, reaching groove III	Domed	Scutal areas unarmed; posterior margin of dorsal scutum and free tergites with a row of conical, enlarged tubercles	Much wider at apex	Absent
*Eulibitia*	α	Visible only apically in dorsal view, reaching groove III	Depressed medially	Entirely unarmed	Much wider at apex	Absent
*Metalibitia*	γ	Visible only apically in dorsal view, reaching groove IV	Depressed medially	Scutal areas I–III unarmed or with a pair of tubercles; IV with two large tubercles	Almost rectangular	Absent
*Moselabius*	α	Visible only apically in dorsal view; reaching groove IV	Depressed medially	Posterior margin of dorsal scutum and free tergites with a pair of enlarged tubercles	?	Present
*Syncynorta*	α	?	Domed	Scutal areas I–IV and posterior margin of dorsal scutum with a pair of enlarged tubercles; free tergites with row of large tubercles	Rectangular	?

## Supplementary Material

XML Treatment for Platygyndes

XML Treatment for Platygyndes titicaca
